# Effectiveness of Action to Reduce Exposure of Free-Ranging California Condors in Arizona and Utah to Lead from Spent Ammunition

**DOI:** 10.1371/journal.pone.0004022

**Published:** 2008-12-24

**Authors:** Rhys E. Green, W. Grainger Hunt, Christopher N. Parish, Ian Newton

**Affiliations:** 1 Department of Zoology, Conservation Science Group, University of Cambridge, Cambridge, United Kingdom; 2 Royal Society for the Protection of Birds, The Lodge, Sandy, Bedfordshire, United Kingdom; 3 The Peregrine Fund, Boise, Idaho, United States of America; 4 Centre for Ecology and Hydrology, Monks Wood Experimental Station, Abbots Ripton, Huntingdon, United Kingdom; University of Oxford, United Kingdom

## Abstract

California condors (*Gymnogyps californianus*) released into the wild in Arizona ranged widely in Arizona and Utah. Previous studies have shown that the blood lead concentrations of many of the birds rise because of ingestion of spent lead ammunition. Condors were routinely recaptured and treated to reduce their lead levels as necessary but, even so, several died from lead poisoning. We used tracking data from VHF and satellite tags, together with the results of routine testing of blood lead concentrations, to estimate daily changes in blood lead level in relation to the location of each bird. The mean daily increment in blood lead concentration depended upon both the location of the bird and the time of year. Birds that spent time during the deer hunting season in two areas in which deer were shot with lead ammunition (Kaibab Plateau (Arizona) and Zion (Utah)) were especially likely to have high blood lead levels. The influence upon blood lead level of presence in a particular area declined with time elapsed since the bird was last there. We estimated the daily blood lead level for each bird and its influence upon daily mortality rate from lead poisoning. Condors with high blood lead over a protracted period were much more likely to die than birds with low blood lead or short-term elevation. We simulated the effect of ending the existing lead exposure reduction measures at Kaibab Plateau, which encourage the voluntary use of non-lead ammunition and removal of gut piles of deer and elk killed using lead ammunition. The estimated mortality rate due to lead in the absence of this program was sufficiently high that the condor population would be expected to decline rapidly. The extension of the existing lead reduction program to cover Zion (Utah), as well as the Kaibab plateau, would be expected to reduce mortality caused by lead substantially and allow the condor population to increase.

## Introduction

The California Condor (*Gymnogyps californianus*) became extinct in the wild in the 1987 when the last wild individual was captured and added to the captive flock, which then consisted of 27 birds. Since 1992, releases of these birds and their captive-bred progeny have re-established wild populations of condors in California, Mexico and around the Grand Canyon in Arizona and Utah. Individual condors in these populations have suffered from lead poisoning caused by ingested ammunition, which is the most frequently diagnosed cause of death among Grand Canyon condors. This holds despite intensive efforts to monitor blood concentrations of lead and to treat birds with high levels using chelating agents [1]. The condors in the Grand Canyon population range widely in Arizona and Utah and feed on carrion, a proportion of which comes from the carcasses of game animals shot by hunters using lead ammunition. Ingestion of shotgun pellets and fragments of bullets in flesh from such carcasses is the route by which lead poisoning occurs. Condors are located as frequently as possible using satellite tags and VHF radio tags and those that cease to move are recovered. Birds are also captured routinely and their blood lead concentrations measured. Any individuals with high levels are held for treatment to reduce the burden of lead in the body before release. Action is also taken on the Kaibab Plateau, Arizona to reduce exposure of condors to lead by encouraging hunters to use non-lead bullets and to remove potentially contaminated gut piles. The level of condor mortality caused by lead that would occur in the absence of chelation therapy and lead exposure reduction is of interest because it might not always be practical to locate birds daily and trap all condors routinely once or twice per year for blood lead monitoring, and implementation of lead exposure reduction schemes requires resources [2]. Could the reintroduced population persist if the lead exposure reduction and treatment programs ceased or were reduced in scope? What would be the effect of reducing exposure to spent lead ammunition throughout the range of this population? As a step towards addressing these questions, we report here a statistical model of blood lead levels in free-ranging condors, which extends previous analyses [3]. We took advantage of the unusually complete radio-tracking data, which allow the influence on blood lead of the location of condors within their geographical range to be assessed. Our objectives were to model the distribution of blood lead levels throughout the year in the absence of treatment, and then to estimate the mortality rates that would prevail. Finally, we used the model to explore the possible effects on condor mortality of withdrawing or increasing measures to reduce exposure of condors to spent lead ammunition.

## Materials and Methods

### Field studies

We used data for 2005, 2006 and 2007 derived from the monitoring of movements and blood lead levels of free-ranging condors [1]. The dependent variable in our analyses was the concentration of lead in the blood of a condor determined within five days after capture. Blood lead levels were determined using a portable field tester (LeadCare Blood Lead Testing System). Some blood samples were also analysed by atomic absorption spectroscopy at the Louisiana State University Diagnostics Laboratory using a Perkin Elmer Analyst 800. Levels of lead in the same blood sample measured using the field tester and in the laboratory were strongly correlated, but laboratory measurements gave significantly higher values (see Figure 2 of reference [1]). Using 99 cases in which the lead concentration in the same blood sample had been determined by both methods, we found that the mean concentration of lead measured in the laboratory was larger than that from the field tester by a factor of 1.914. In all analyses we therefore used a laboratory determination whenever one was available and otherwise adjusted the field tester measurement using this correction factor.

We modeled the blood lead level in each free-ranging condor in relation to the locations it had used before it was recaptured for testing. During the study period, roost locations of condors marked with VHF or satellite tags were determined on the majority of days for all tagged condors, and attributed to one of the following five zones; Paria (Vermilion Cliffs), Colorado River Corridor, Kaibab Plateau, South Zone and North Zone (Utah). A location was taken to be a roost location if it was obtained later than16.00h. local time. Condors are known to range widely, even within a day [3], so the ideal analysis would take into account the bird's location at several times during each day. However, only the data for satellite tagged birds would permit this. Roost locations were recorded for as many days as possible during the period beginning with the initial release of each bird, or its release after capture for blood lead monitoring and ending with another capture at which blood lead concentration was determined. For days on which the roost location was not recorded, we interpolated the roost zone used by assuming that it was the same as that on the nearest day with data available. Overall, it was necessary to interpolate the roost zone on 27.2% of days, with the range of this proportion for individual birds being 11.1% to 59.9%. We had eligible data derived from 60 individual condors consisting of 322 pairs of blood lead measurements preceded by periods comprising, in total, 41,230 bird-days with known or interpolated roost locations.

Numbers of deer, elk and buffalo reported as killed by hunters in each zone in 2005–2007 were obtained from the Arizona Game and Fish Department and the Utah Department of Natural Resources. We estimated the number of carcasses and gut piles potentially contaminated with lead and left in the field for scavengers by using information collected on the proportion of kills made with lead ammunition and the number of lead-killed animals from which gut piles were brought in by hunters for safe disposal. We also assumed that in addition to the number of animals reported as killed with lead bullets, an additional 10% of that number were wounded and died unrecovered soon after, thereby becoming available to condors.

### Analysis and statistical modeling of blood lead data

**Figure 1:**
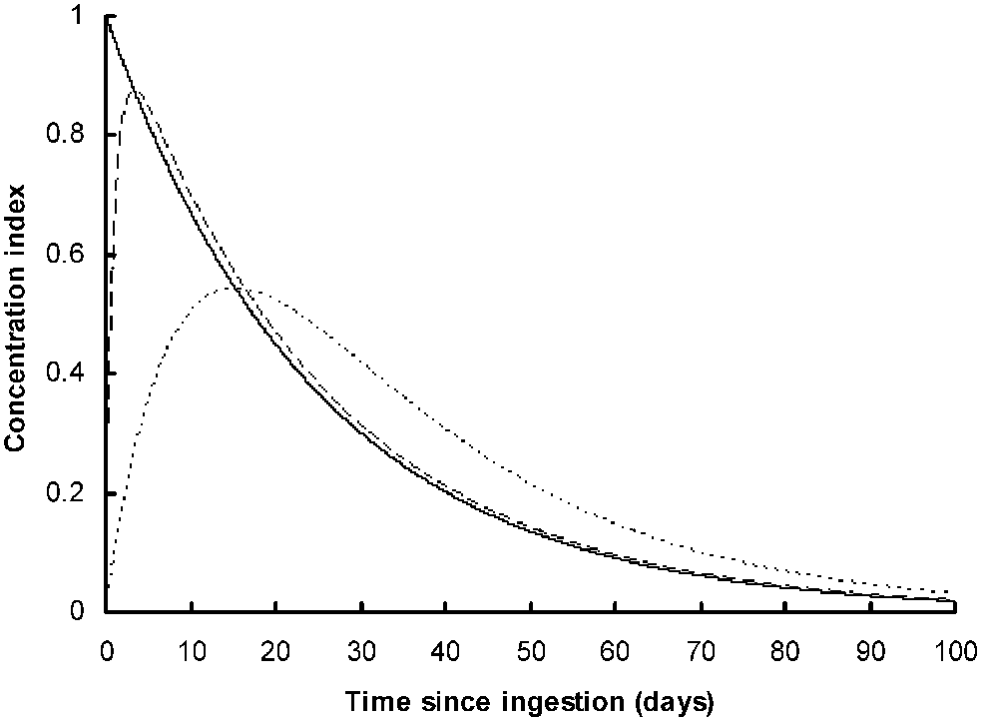
Models of the relationship between blood lead concentration and time since ingestion of metallic lead in California condors. The schematic diagram shows the family of models (see text) assumed to describe changes in blood lead concentration in untreated condors in relation to time since ingestion, in the absence of any further ingestion. The value of model parameter *k_2_* is 0.04 for all three curves, but *k_1_* is very large for the solid line, *k_1_* = 1 for the dashed line, and *k_1_* = 0.1 for the dotted line.

We assumed that, with no further ingestion, the relationship between blood lead concentration and time after ingestion of fragments of metallic lead could be described by a simple three compartment model, with one-way movement of lead between successive pairs of compartments. Although this model is a simplification, it has the advantage of requiring the estimation of only two parameters and seems likely to capture the main features of real changes in blood lead. We assumed that a constant proportion of the ingested lead enters the blood from the gut per unit time and that fragments are not expelled from the gut within the period that significant absorption is occurring. Hence, the proportion of the lead ingested that remains in the gut at time *t* (in days) since ingestion is given by exp(−*k_1_t*), where *k_1_* is a constant, and (1−exp(−*k_1_t*)) is the proportion of lead ingested that has moved from the gut to the blood by that time. We also assumed that a constant proportion per unit time of the lead present in the blood was lost to another compartment, such that the amount in the blood would decline by a proportion (1−exp(−*k_2_*)) per day in the absence of absorption. The quantity of lead in the blood, as a proportion of that ingested, is then given by the function
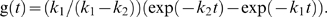
(1)Assuming that blood volume is constant, blood lead concentration is proportional to g(*t*). Note that this expression approximates to g(*t*) = exp(−*k_2_t*) when *k_1_* is much larger than *k_2_*. That is, when absorption from the gut is very rapid, blood lead concentration declines exponentially with time since ingestion. The model is illustrated for a single value of *k_2_* and three values of *k_1_* in Fig. 1.

**Figure 2:**
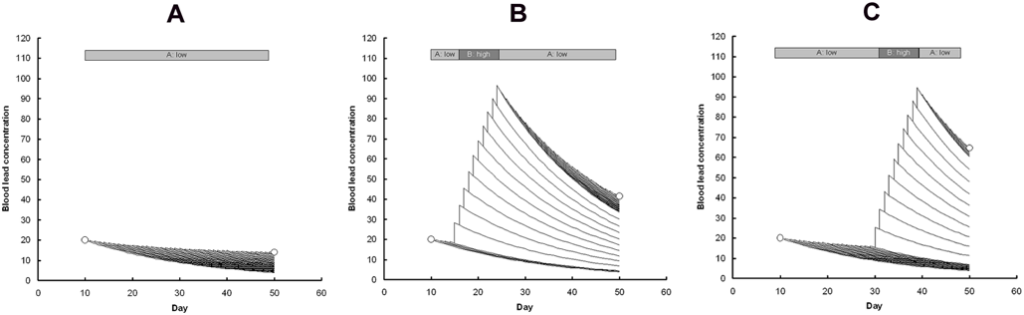
Hypothetical changes in blood lead concentration over time for California condors moving between zones with high and low daily risk of ingesting lead. The schematic diagram shows average hypothetical changes in blood lead concentration (µg dL^−1^) for condors moving between zones with high and low daily risk of ingesting lead. Curves show the time course of components of blood lead derived from each day's intake. Birds remaining in the low risk zone (A) throughout would be expected to show a decline in blood lead over the time between a pair of measurements (open circles). Birds visiting the high risk zone early in the interval between measurements (B) would be expected to show an increase in blood lead concentration. Birds visiting the high risk zone for the same number of days, but later in the interval (C), would be expected to show the highest increase in blood lead concentration.

We next used the function g(*t*) to explore how the concentration of lead in the blood of an average condor would be expected to change over time, given the possibility of ingestion of lead on more than one day. We assumed that the condor spends some time in areas where there is a high risk each day of ingesting lead and some time in low risk areas. We imagined a large number of condors, all showing the same movement pattern. On each successive day, the average quantity of lead ingested by the birds would, if it was all absorbed immediately, increase the average concentration of lead in the blood by an amount *m*, which we call the mean daily blood lead increment. In fact, we would expect the component of the concentration of lead in the blood derived from the lead ingested on a given day to be given by *m* g(*t*), where *t* is the time elapsed since that day. How the lead ingested on successive days would influence the total concentration of lead in the blood can be visualised with the aid of the schematic diagrams shown in Fig. 2. For simplicity, we use g(*t*) = exp(−*k_2_t*) in this illustration, but the equivalent for the function given by Eq.(1) can easily be envisaged. Consider first a sample of birds that remain in an area with a low risk of ingesting lead (area A). If the average amount of lead ingested per day is small relative to the rate at which it is eliminated from the blood, we would expect that the average blood lead concentration would decline over time (Fig. 2(a)). Hence, we would expect that the average of measurements of blood lead concentration at the end of a period in which the condors had remained in a low risk area would be lower than the average of measurements made at the beginning of the period (shown in the diagram by circles). However, if the condors instead spend part of the period in a high risk area (area B) and the rest in A, we would expect that the average blood lead concentration would be higher at the second measurement than at the first (Fig. 2(b)). Furthermore, the timing of the birds' visit to the high risk area would be expected to influence the change in blood lead between the two measurements. In this case, a visit to area A late in the interval between two blood lead measurements (Fig. 2(c)) is expected to result in a larger increase in blood lead than a visit of the same duration earlier in the interval (Fig. 2(b)). Note that this effect of the timing of the visit to area A might be different if g(*t*) was of the form that the blood lead concentration from a given day's ingested lead first increased and then decreased (as in Fig. 1). The pattern of variation in changes over time in blood lead shown by these illustrations suggests that, given sufficient pairs of observations of blood lead concentration for condors whose location was known during the intervening period, it would be possible to estimate the parameters of g(*t*) and the mean daily blood lead increment for different zones.

It is evident from Fig. 2 that the average blood lead measurement *v_s_* at time *t_s_* is given by
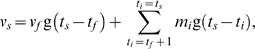
(2)where *v_f_* is the previous blood lead measurement at time *t_f_*, and *m_i_* is the mean blood lead increment for the location where the bird was on day *t_i_* of the period intervening between *t_f_* and *t_s_*. Applying this model to real data on pairs of blood lead measurements from condors whose location was known during the intervening period, we used Eq.(2) to calculate E(*v_s_*), the expected value of *v_s_*, from the observed value *v_f_* and provisional starting values of the parameters *k_1_*, *k_2_* and *m_i_*. The values of the *m_i_* were assumed to be specific to each zone used by the condors and to be different between the hunting season and outside the hunting season. Hence, there were 10 values of *m_i_*, two for each of the five zones. For each observation, we then calculated the log-likelihood of observing blood lead level *v_s_*, given the expected value E(*v_s_*), assuming that observed values were distributed log-normally around the expected mean value with variance *s* ^2^. We summed these log-likelihoods to give the total log-likelihood of the data under the model. We then used a simplex procedure to find the values of the parameters *k_1_*, *k_2_*, *m_i_*. and *s* which maximised the log-likelihood. Because the logic underlying our model does not permit the mean daily blood lead increment to be negative, estimated values of *m_i_* were constrained to exceed zero using log transformation. Some *v_s_* values measured only with the field tester were known to exceed its upper limit of quantification, but the actual values were unknown. These were treated as right-censored observations exceeding the upper limit of quantification in the likelihood calculation [4]. Confidence limits for parameter estimates were obtained by bootstrap sampling, with replacement, from the actual data, with individual condors as bootstrap sampling units. Sets of parameter estimates were obtained by the maximum-likelihood method described above for each of 1,000 bootstrap samples and the central 950 of the estimates of each parameter were used to define its 95% confidence interval.

### Analysis of mortality caused by lead intoxication in relation to movements

We wished to estimate the daily probability of a condor dying from lead poisoning as a function of its recent history of movement among zones in a way that would be likely to reflect its exposure to lead. It was not practical to model the daily probability of death from lead poisoning as a function of recent presence in all of the five zones within and outside the hunting season. This is because only eight deaths from lead poisoning were observed in 2005–2007 and, of these, two deaths occurred early in 2005 with inadequate data on their prior movement history, leaving only six birds with sufficient information. A model with many parameters cannot be supported by this small sample size. Therefore, we undertook the modeling of mortality in two steps. First, we used the zone- and season-specific estimates of mean daily blood lead increments *m* from the analysis described above and the observed movements of each bird to reconstruct expected values E(*v*) of blood lead concentration from Eq.(2) for every day on which each bird was free-ranging. Next, we took a weighted mean of the E(*v*) on days up to and including each focal day on which the bird was free-ranging. We considered it necessary to use in the model the weighted mean of E(*v*) on the focal day and a set of previous days, rather than just E(*v*) on the focal day itself, because it seemed biologically realistic for the probability of death to be determined by a bird's recent history of blood lead concentration. We used a weighting function, so that the weight for a given day *t_i_* on or previous to the focal day t* was exp(−(t*−*t_i_*)^2^/*q* ^2^), where *q* is a constant. We used logistic regression to fit the relationship between the daily probability of death from lead poisoning and the weighted mean of E(*v*) on and prior to each focal day. We used a bisection search to determine the value of the parameter *q* of the weighting function that maximised the log-likelihood of the data. The data to which the model was fitted comprised all bird-days in 2005–2007 on which condors were free-ranging, including the two bird-days on which deaths from lead poisoning of free-living birds occurred and four bird-days on which birds were taken into captivity with high blood lead levels from which they subsequently died. We obtained confidence limits of the parameters of the logistic regression and of *q* by a bootstrap procedure. We drew a bootstrap sample, with replacement, from the data contributed by the 60 individual condors, with individual birds as bootstrap units. From this sample we estimated the *m* and *k* values as described above, reconstructed the E(*v*), and then estimated *q* and the logistic regression parameters. We took 1,000 such bootstrap samples and took the central 950 estimates for each parameter as its 95% confidence interval.

### Analysis of movements among zones

We wished to use information on movement patterns to simulate condor mortality from lead intoxication in the absence of intervention to remove condors from the wild and to treat those with high blood lead levels. Clearly, we could not use the observed movement pattern directly for this because runs of days in particular zones that would otherwise have occurred were disrupted by capture and removal from the wild. We therefore made a statistical model, based upon the observed sequences of movement among zones, to describe the probability that a condor present in a given zone on one day (the origin zone) would move to another specified zone (the destination zone) on the next day, rather than remaining in the origin zone or moving elsewhere. Inspection of the data on observed and interpolated roost locations for 2005–2007 indicated that there was an annual cycle in the use made of the different zones. Hence, we fitted a model in which the logit of the daily probability of a condor moving between two specified zones was a sinusoidal function of time of year *z* (the date expressed as a proportion of the calendar year). We also wished to allow the probability of movement to be free to vary systematically with time across the whole three year period. We therefore also included a quadratic function relating the logit probability of movement to the time, in years *y* elapsed since 31 December 2004. The expression used was
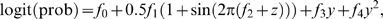
(3)where the *f* are constants. We estimated the parameters of this logistic regression model using a maximum-likelihood method.

### Estimating daily blood lead concentrations

We used a Monte Carlo process, together with the statistical model of movements among zones described above, to simulate movements of condors among zones for the three-year period 2005–2007. For each of 10,000 simulated condors, we generated a random number on each successive day. We used it and the probabilities for that day of movement from the origin zone to each of the four destination zones, obtained from Eq.(3), to determine which zone each bird moved to, or whether it remained within the origin zone. The origin zone on the first simulated day was selected by generating a random number and using the observed proportions of known locations of birds on 1 January 2005 to allocate the bird to a starting zone. We then ran the model for one year using 2005 dates as a burn-in procedure and then continued to run the model for a further three years, starting again from 1 January 2005; only this latter period being used further. We summarized the simulated movements by calculating the proportion of simulated condors in each zone on each day of the three-year period.

For each simulated condor, we also estimated its expected blood lead concentration on each day by allowing the lead present on the previous day to change according to the function g(*t*), with maximum-likelihood estimates of its parameters, and by adding the mean daily blood lead increment expected for the zone and season, again using the maximum-likelihood estimates. We calculated the geometric mean and the variance of the simulated blood lead concentrations on each day. The variance was obtained by adding together the variance of the modeled blood lead levels calculated across simulated individuals and fitted value of the residual variance *s* ^2^ from the model of blood lead concentration in relation to zone (see above). Proportions of simulated condors in different categories of blood lead concentration on each day were then calculated from the geometric mean and variance. Categories were defined according to Franson [5] and Fisher *et al.* [6].

### Estimating mortality caused by lead intoxication

We also used the Monte Carlo model of condor movements and blood lead levels, described above, to estimate the mortality rate caused by lead intoxication. On each successive day of the simulated sequence, we calculated the weighted mean expected blood lead level, using values of E(*v*) for the focal day and previous days and the weighting function, with the maximum-likelihood value of *q*. We then used the maximum-likelihood values of the parameters of the logistic regression relating the daily probability of death to weighted blood lead level to calculate the expected probability of death. A random number between zero and one was generated and, if it was less than the expected value of the probability of death, the condor was simulated to have died from lead poisoning. The simulation for that bird was then terminated. The procedure was repeated for all 10,000 simulated condors. From the set of simulations, we calculated the proportion of the cohort of birds present on 1 January 2005 that had not yet died from lead poisoning on each successive day until the end of 2007. This gave a simulated survivorship curve, ignoring mortality from other causes. We also obtained the proportion of birds simulated as not having died from lead intoxication at the end of the three years. We raised this proportion to the power 1/3 and then subtracted the result from one to give the simulated average annual mortality rate from lead poisoning.

We ran the Monte Carlo simulations for each of the bootstrap sets of parameter values described in the analysis of mortality and took the central 95% of bootstrap estimates of annual mortality caused by lead poisoning to be its 95% confidence limits.

## Results

### Relationship of blood lead level to time since ingestion

We fitted the model summarized in Eq.(2) to the 322 pairs of blood lead measurements derived from 60 condors, as described above. We used both the one-parameter and two-parameter forms of the function g(*t*) that relates blood lead concentration to time since ingestion. The one-parameter version of g(*t*) gave a higher log-likelihood and we therefore selected it for reasons of parsimony. The maximum-likelihood value of the parameter *k_2_* was 0.0408 (95% confidence limits 0.0286–0.0581). This function describes an exponential decline in blood lead concentration with a half-life of 17.0 days (95% confidence limits 11.9–24.2 days).

### Mean daily blood lead increment in relation to zone and season

**Figure 3:**
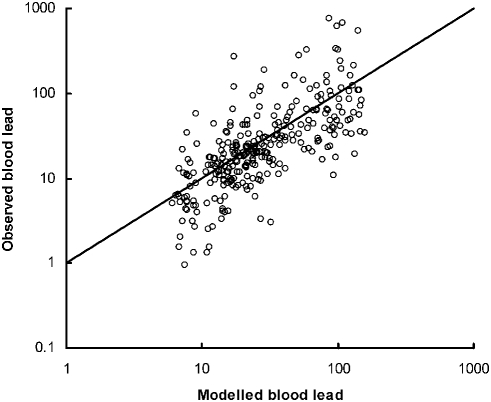
Relationship between observed and modeled blood lead concentration (µg dL^−1^) for 296 measurements on free-living California condors 2005–2007. The model is that described in the text with maximum-likelihood parameter estimates given in Table 1. Measurements that exceeded the limit of quantification of the field tester and were not duplicated by a laboratory measurement were excluded. The line shows the expected relationship if the observed and modeled measurements were identical.

The maximum-likelihood estimates of mean daily blood lead increment *m* from the fitted model summarized in Eq.(2), with the one-parameter version of g(*t*), are shown in Table 1. The fitted model performed well in accounting for variation in blood lead concentration. There was a high correlation between log-transformed observed and modeled blood lead values (*r* = 0.708) and deviations from the expected values were approximately uniform across the range of expected values (Fig. 3).

**Table 1:** Maximum-likelihood estimates of mean daily increment of blood lead concentration (µg dL^−1^ d^−1^) for free-ranging California condors, with 95% bootstrap confidence limits, outside and within the hunting seasons of 2005–2007, for five zones.

	Mean daily increment	95% Confidence limits			Reported kills per day	Lead contaminated remains per day
*Outside hunting season*						
Colorado River Corridor	1.21	0.76	-	1.93	0.00	0.00
Kaibab Plateau	0.79	0.40	-	1.56	0.04	0.02
Paria	0.27	0.16	-	0.46	0.00	0.00
South Zone	1.29	0.71	-	2.32	0.00	0.00
North Zone	1.26	0.77	-	2.05	0.00	0.00
*Hunting season*						
Colorado River Corridor	0.00	0.00	-	0.92	0.35	0.16
Kaibab Plateau	15.36	8.00	-	29.51	36.41	14.76
Paria	0.24	0.05	-	1.19	0.08	0.04
South Zone	2.13	0.00	-	8.27	0.00	0.00
North Zone	14.15	5.75	-	34.81	40.11	41.23

Also shown are the reported numbers of deer, elk and buffalo killed per day within and outside the deer and elk hunting seasons in 2005–2007, and the number of these per day (including additional wounding losses of deer and elk) left as lead contaminated carcasses or gut piles. Small numbers of buffalo killed in Kaibab in March–August were included in the non-hunting season total.

The estimates of mean daily blood lead increment were low (<3 µg dL^−1^ d^−1^) for most zones and seasons, but strikingly higher (>14 µg dL^−1^ d^−1^) in the Kaibab and North zones during the hunting season. The daily blood lead increment was much lower outside the hunting season than within it for both the Kaibab and North zones, with the difference between hunting and non-hunting seasons being smaller and inconsistent in direction for the other zones (Table 1).

The statistics collected on hunting showed that the average number of large game animals killed per day of the season was much higher during the hunting seasons in Kaibab and North zones than outside the hunting season in these zones, and also higher than for all other zones, both within and outside the hunting season (Table 1). The estimated number of lead-contaminated carcasses and gut piles produced per day of the season, after allowing for the effects of the program to encourage the use of copper bullets and to remove contaminated gut piles, was also higher in Kaibab and North zones during the hunting season than in other zones and seasons. However, the lead reduction program in Kaibab resulted in estimated lead exposure being lower during the hunting season in Kaibab than in North zone, despite the numbers of kills per day being similar in both zones.

### Mortality caused by lead intoxication in relation to blood lead concentration reconstructed using movement history

**Figure 4:**
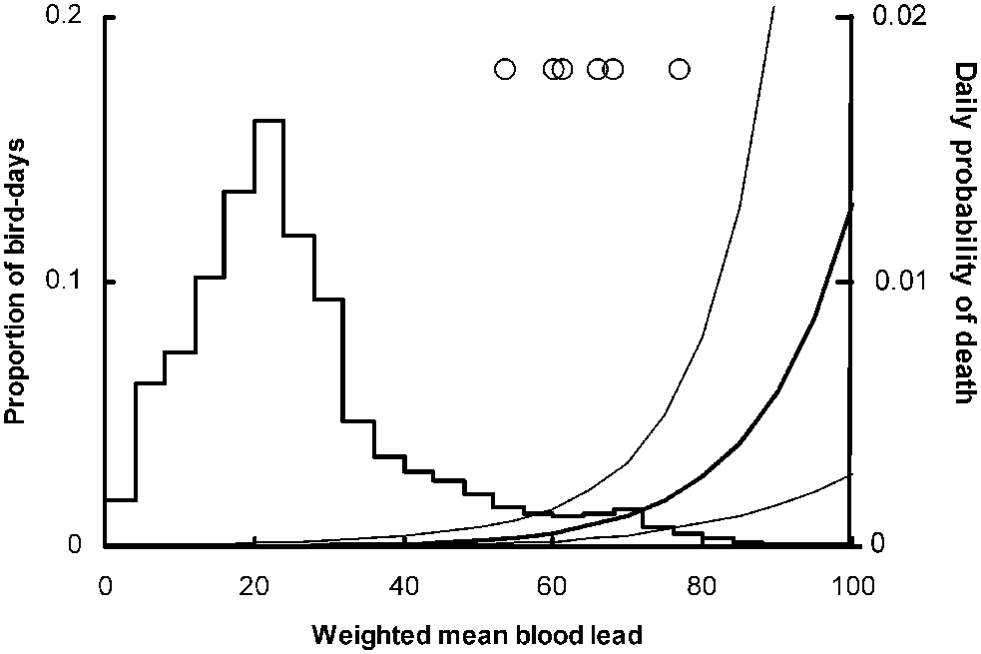
Daily mortality rate of California condors from lead poisoning in relation to the weighted mean of reconstructed blood lead concentration (µg dL^−1^) prior to the focal day. The histogram (left-hand scale) shows the distribution of weighted mean blood lead values on days when monitored condors did not die from lead poisoning. Circles show values on the day of death or last capture for the six birds that did die from lead poisoning. The thick curve shows the logistic regression model, fitted to these data, relating daily probability of death (right-hand scale) to the weighted mean of modeled blood lead concentration. Thin curves show 95% bootstrap confidence limits.

Although only six condors with sufficient data for analysis died from lead intoxication during our study period, there was a marked difference between the blood lead concentration history of these birds, reconstructed using information on their movements, and the equivalent results for birds that did not die from lead intoxication. Simulated blood lead concentration was higher on the day of death or final capture of the birds that died, and for a substantial period beforehand, compared with days upon which death from lead intoxication did not occur. This difference was greatest when a value of 125.3 days was chosen for the parameter *q*, which is used in calculating the weighted mean concentration over the days prior to the focal day (Fig. 4). Other parameter values of the fitted logistic regression model that relates the daily probability of death to the weighted mean reconstructed blood lead concentration are given in Table 2.

**Table 2:** Maximum-likelihood estimates, with 95% bootstrap confidence limits, for the parameters of a logistic regression model relating the daily probability of death of a California condor from lead poisoning to the weighted mean of its modeled blood lead concentration (µg dL^−1^) on the focal day and on previous days.

Parameter	Estimate	Lower C.L.	Upper C.L.
*q*	125.3	48.5	323.1
Intercept	−12.31	−14.80	−9.81
Slope	0.07971	0.04460	0.1148

The parameter *q* (in days) determines the shape of the weighting function used to calculate the weighted mean blood lead concentration.

### Movements among zones

**Figure 5:**
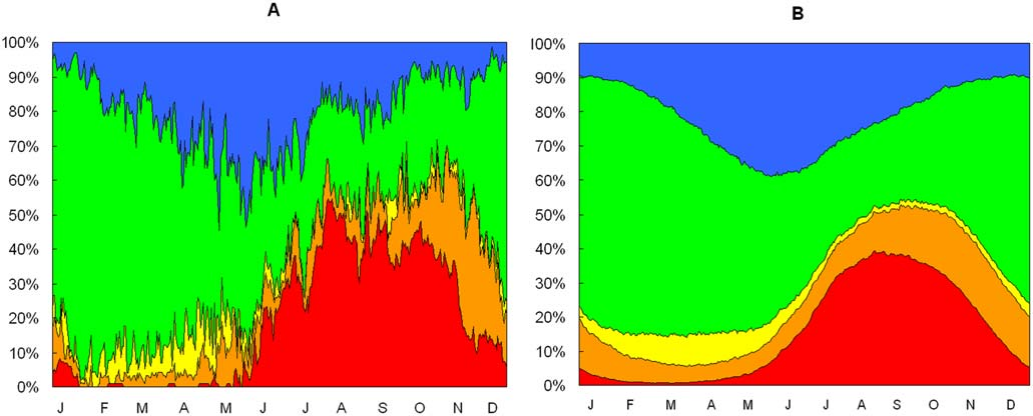
Movements of California condors among zones. (A) Observed proportions of free-ranging condors in each of five zones (red = North Zone, orange = Kaibab Plateau, yellow = Colorado River Corridor, green = Paria, blue = South Zone) in relation to time of year for observations pooled over the three-year period 2005–2007. (B) Modeled proportions of condors in the same zones from the Markov chain model described in the text.

The parameter estimates of the logistic regression models relating the log-transformed probabilities of movements between each pair of origin and destination zones to year and time of year are shown in Table 3. The proportions of birds simulated as present in the five zones are shown in relation to time of year, with results for the three simulated years pooled, in Fig. 5(b). The pattern of change through the year in the proportions of birds in each zone resembles that in the raw roost location data (Fig. 5(a)). The Kaibab Plateau and North zones were most used from July to November. The Paria and Colorado River zones were most used from December to April. The South zone was most used from February to May.

**Table 3:** Fitted values of parameters of a Markov chain model (see text) relating the daily probability of a tagged California condor moving from a zone in which it was initially present (origin) to a given other zone (destination) to season and year.

Origin zone	Destination zone	*f_0_*	*f_1_*	*f_2_*	*f_3_*	*f_4_*
Colorado River	Kaibab Plateau	−2.250	−2.284	0.212	−2.714	0.986
Colorado River	Paria	−0.732	−0.905	0.408	1.356	−0.593
Colorado River	South Zone	−1.178	−3.254	0.237	−1.417	0.461
Colorado River	North Zone	−25.324	−6.807	0.146	22.813	−5.309
Kaibab Plateau	Colorado River	−2.267	−2.706	0.347	−3.693	1.291
Kaibab Plateau	Paria	−0.408	−1.060	0.377	−0.628	0.094
Kaibab Plateau	South Zone	−1.110	−1.719	0.221	−0.577	0.137
Kaibab Plateau	North Zone	−2.126	−2.674	0.177	−0.323	0.147
Paria	Colorado River	−3.348	−2.204	0.422	1.412	−0.478
Paria	Kaibab Plateau	−3.031	−1.882	0.118	0.747	−0.186
Paria	South Zone	−1.311	−2.966	0.286	−0.072	−0.071
Paria	North Zone	−4.107	−3.967	0.108	1.047	−0.168
South Zone	Colorado River	−5.322	−0.770	0.363	0.390	−0.109
South Zone	Kaibab Plateau	−4.269	1.721	0.463	0.857	−0.313
South Zone	Paria	−0.914	−1.288	0.351	0.131	−0.157
South Zone	North Zone	−4.215	−4.224	0.101	0.692	−0.032
North Zone	Colorado River	−15.185	−0.130	0.041	9.266	−2.042
North Zone	Kaibab Plateau	−4.278	2.494	0.049	−0.203	0.061
North Zone	Paria	−1.678	−1.676	0.487	−0.625	0.118
North Zone	South Zone	−4.763	−1.566	0.382	1.060	−0.190

### Estimated daily blood lead concentration

**Figure 6:**
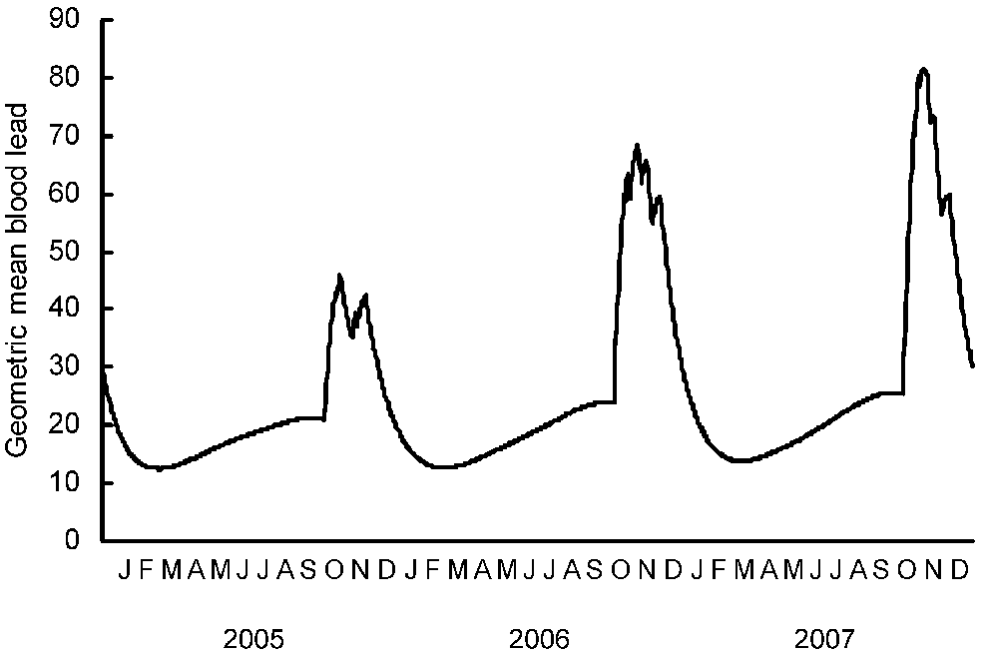
Modeled geometric mean blood lead concentration (µg dL^−1^) of free-ranging California condors for the three years 2005 to 2007.

**Figure 7:**
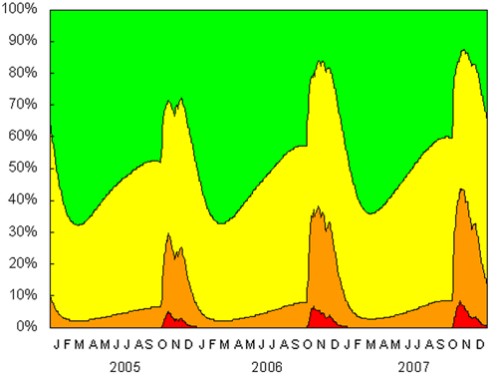
Proportions of California condors with lethal (red, >500 µg dL^−1^), toxic (orange, >100 µg dL^−1^), subclinical (yellow, >20 µg dL^−1^) and normal (green, <20 µg dL^−1^) blood lead concentration, as reconstructed from Markov chain simulated movement patterns for the three-year period 2005–2007.

The reconstructions of blood lead concentrations for the period 2005–2007 showed large peaks in geometric mean concentrations in November–December of each year (Fig. 6). Measured over the whole three-year period, blood lead concentrations exceeded the upper bound of the normal range on about half of the simulated condor-days. There was a peak in the proportion of birds simulated as having lethal lead levels in November of each year, which reached 5.1, 6.3 and 8.5% of condors in 2005, 2006 and 2007 respectively (Fig. 7).

### Mortality caused by lead intoxication in the absence of chelation therapy

**Figure 8:**
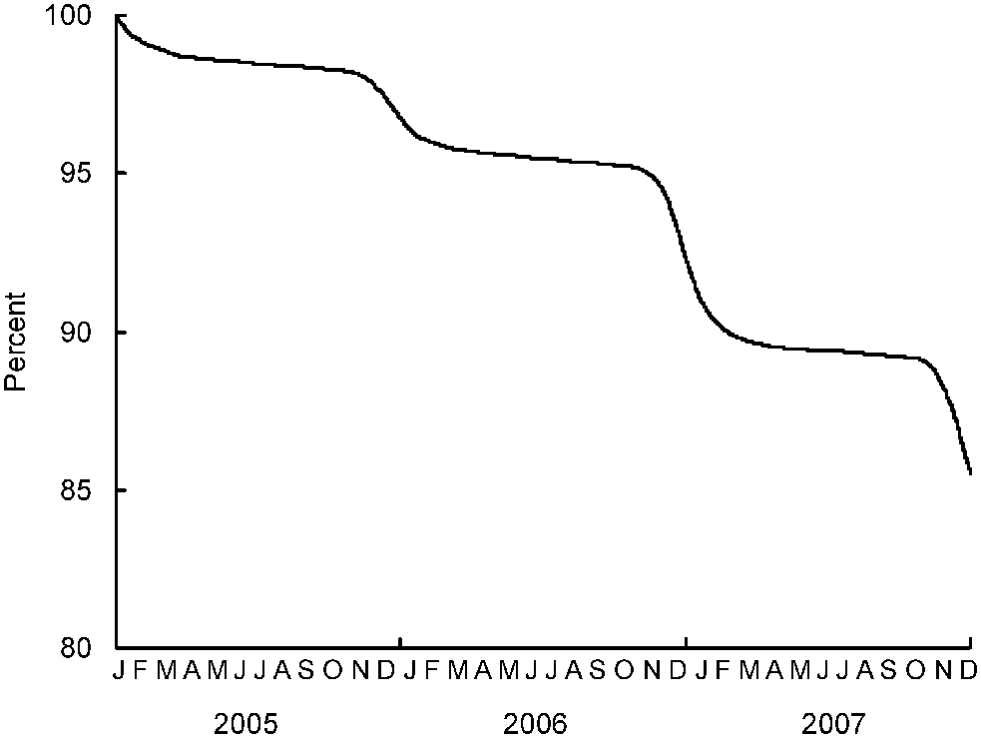
Modeled percentage of a hypothetical cohort of California condors alive on 1 January 2005 which had not yet died from lead poisoning on each day of the ensuing three-year period. It was assumed that birds were only subject to mortality caused by spent lead ammunition under the current exposure scenario, and did not receive chelation therapy.

The estimated proportion of a cohort of condors present on 1 January 2005 which had yet to die from lead intoxication showed three periods of rapid decline in November–January in each of the three years (Fig. 8). Over the whole three-year period, the simulations showed that 14.5% of condors present at the start of the period had died from lead intoxication by the end of it. This is equivalent to an annual probability of dying from lead intoxication of 5.1% (Table 4).

**Table 4:** Modeled proportions of California condors dying from lead poisoning during the period 2005–2007, and equivalent annual death rates, under various scenarios for the reduction of exposure to spent lead ammunition.

	Three-year rate	Annual rate	95% confidence limits of annual rate		
As now	0.1450	0.0509	0.0146	-	0.1519
No reduction program	0.5002	0.2064	0.0428	-	0.5029
UT reduction as in AZ now	0.0458	0.0155	0.0048	-	0.0479

The scenarios represent the 2005–2007 pattern of exposure (“As now”), the recent pattern, but with the effects of the program to reduce lead exposure at Kaibab Plateau (Arizona - AZ) removed (“No reduction program”), and with the average proportion of reduction of lead exposure at achieved at Kaibab plateau in 2005–2007 applied to both Kaibab Plateau and North zones (Utah – UT)- this scenario is called “UT reduction as in AZ now”).

### Effect upon mortality caused by lead intoxication of ending the program to reduce exposure to lead or increasing the area covered by the program

We simulated the effect upon mortality of the existing program to reduce the exposure of condors to lead by changing the parameter value for *m*, which specifies the mean daily blood lead increment for each zone and season. Over the three hunting seasons 2005–2007, it was estimated that the program to reduce exposure to lead in the Kaibab zone diminished the number of potentially lead-contaminated carcasses and gut piles remaining for scavengers by 63%, compared with what would otherwise have been present. Hence, we multiplied the estimated value of *m* for the Kaibab zone during the hunting season in Table 1 by a factor of 2.7 ( = 1/(1−0.63)) to simulate what would have occurred without the lead reduction program. Simulations of mortality caused by lead in the absence of the Kaibab reduction program indicate a much higher mortality; a 50% rate in three years, which is equivalent to an annual mortality rate of 20.6% (Table 4).

We also simulated the effect of implementing a lead reduction program in the North zone (Utah) with similar effectiveness to the existing program in the Kaibab zone. To do this, we used the value of *m* for the Kaibab zone in the hunting season shown in Table 1 (i.e. the value obtained with the existing program in place), but we divided the value of *m* in Table 1 for the North zone in the hunting season by 2.7. Simulations of mortality caused by lead with lead reduction programs in both Kaibab and North zones indicate lower mortality; a 4.6% mortality rate in three years, which is equivalent to an annual rate of 1.6% (Table 4).

## Discussion

Our analysis of changes in blood lead concentration between release and recapture reveals a striking tendency for condors to run a high risk of acquiring elevated blood lead concentrations when they visited the Kaibab and North zones during the hunting seasons for deer and elk in 2005–2007. These two zones are those in which the largest numbers of these quarry species were hunted. A previous analysis [3], using data for July 2001 to June 2005, also showed that condors that visited the Kaibab zone were more likely to acquire high blood lead levels upon recapture than those that did not. Our analysis differs in also finding a high risk when condors visited the North zone in Utah. The difference probably arises because our present analysis includes more recent data (2005–2007) and condors greatly increased their use of the North zone from 2004. Taken together with evidence of shotgun pellets and bullet fragments in recaptured condors with high blood lead levels [1] and observations of condors at carcasses of hunter-killed deer and elk [3], our results indicate that ingestion of contaminated tissue from carcasses and gut piles of game animals killed with lead ammunition in the Kaibab and North zones is the largest source of lead for condors in the Grand Canyon population.

Changes in blood lead levels were affected by the location of the condors over a considerable period prior to sampling. The results were consistent with ingestion of lead being followed, in the absence of further ingestion, by a progressive exponential diminution in its concentration in the blood, with a half-life of 17 days. This pattern is broadly similar to that found in a previous study of captive condors with initially high levels of lead in the blood [7].

The daily death rate from lead intoxication was highest if blood lead levels, simulated using observed movements and the model of lead acquisition and depletion, remained high over a period exceeding one hundred days. Short periods with similarly high blood lead resulted in a lower death rate per day. We think it likely that this reflects a cumulative effect of protracted high blood lead levels on organ function. Condors that visited the Kaibab and North zones less frequently during the hunting season, and which therefore were not simulated as acquiring such high concentrations for such a long period, were much less likely to die from lead intoxication. The Kaibab and North zones were probably especially attractive to condors during the hunting season because of the large quantity of remains of hunted deer and elk available as food there, compared with other parts of the study area.

We used a previously published population model [8] to assess likely long-term trends in the numbers of condors in the absence of further releases and without chelation and other treatment of birds with elevated blood lead concentrations. According to this model, the condor population would tend to decline under present conditions unless natural adult mortality was at the lower end of the likely range or reproduction was at the “maximum conceivable” level (Table 5). Since the assumptions of the “maximum conceivable” scenario are extremely unlikely to apply to any real population of condors, this indicates that the Grand Canyon condor population is unlikely to be self-sustaining at current levels of exposure to lead.

**Table 5:** Annual percentage rates of increase or decrease of a stable age structure model California condor population under various scenarios for the reduction of exposure to spent lead ammunition.

	Non-lead mortality: immature = adult			Non-lead mortality: immature = 2×adult		
	0.90	0.95	0.99	0.90	0.95	0.99
*Most likely*						
No Pb mortality	−0.7	+4.8	+9.2	−4.8	+2.6	+8.8
As now	−5.8	−0.5	+3.7	−9.6	−2.6	+3.2
No reduction	−21.2	−16.8	−13.3	−24.4	−18.6	−13.7
UT reduction as in AZ now	−2.2	+3.2	+7.5	−6.2	+1.0	+7.1
*Maximum conceivable*						
No Pb mortality	+4.4	+10.2	+14.8	−0.8	+7.4	+14.2
As now	−0.9	+4.6	+9.0	−5.9	+2.0	+8.4
No reduction	−17.2	−12.6	−8.9	−21.3	−14.7	−9.3
UT reduction as in AZ now	+2.8	+8.5	+13.0	−2.4	+5.8	+12.5

Details of the scenarios are given in the text and the legend to Table 4. For each scenario, the rate of increase is shown for three plausible annual survival rates for adults in the absence of mortality caused by lead (0.90, 0.95 and 0.99) and for the annual non-lead mortality rate of immatures in the absence of lead being equal to that of adults or twice that of adults. Deaths caused by lead poisoning are assumed to occur in addition to those from other causes. Results are shown for the “Most likely” and “Maximum conceivable” reproductive scenarios [8].

Our simulations indicated a large effect of the existing program to reduce exposure of condors to lead from carcasses and gut piles of deer and elk killed using lead ammunition. At present, this program only covers the Kaibab zone. A simulation of the likely mortality rate due to lead if this program was not in place indicates a very high death rate. A previously published population model [8] indicates that, without releases and chelation therapy, the condor population would decline rapidly under these circumstances, regardless of whether the “most likely” or “maximum conceivable” levels of reproduction are assumed (Table 5). This implies that continuation of the existing lead reduction program in the Kaibab zone, which is conducted by the Arizona Game and Fish Department, is important for the future persistence of the condor population.

Simulation of the extension of the existing lead reduction program to cover the North zone (Utah), as well as the Kaibab Plateau, indicates that this would reduce mortality caused by lead intoxication substantially. With this level of mortality due to lead, the population model indicates that the condor population would increase unless reproduction was at the “most likely” level and adult mortality not caused by lead was at the worst-case (upper) end of the likely range (Table 5). Hence, we recommend that lead reduction programs are implemented as effectively as possible in both the Kaibab and North zones. Only then is a self-sustaining population of free-living California condors likely to persist within the Grand Canyon area currently occupied without continued veterinary management and the regular addition of captive-bred birds.

Poisoning caused by ingestion of spent lead ammunition is a widespread hazard to many species of wild birds [6], but few previous studies have estimated the effect on mortality rates of differential exposure to lead ammunition of free-ranging individuals [9], [10]. Aside from their application to the problem of conserving California condors, we believe that the methods developed for our study may be more widely applicable to other species with large home ranges, within which localized sources of contamination occur.
